# Logic programming reveals alteration of key transcription factors in multiple myeloma

**DOI:** 10.1038/s41598-017-09378-9

**Published:** 2017-08-23

**Authors:** Bertrand Miannay, Stéphane Minvielle, Olivier Roux, Pierre Drouin, Hervé Avet-Loiseau, Catherine Guérin-Charbonnel, Wilfried Gouraud, Michel Attal, Thierry Facon, Nikhil C Munshi, Philippe Moreau, Loïc Campion, Florence Magrangeas, Carito Guziolowski

**Affiliations:** 1 LS2N, UMR 6004, École Centrale de Nantes, Nantes, France; 2 CRCINA, INSERM, CNRS, Université d’Angers, Université de Nantes, Nantes, France; 3 CHU de Nantes, Nantes, France; 4 Unit for Genomics in Myeloma, IUC-Oncopole; and, CRCT INSERM 1037 Toulouse, France; 5 Institut de Cancérologie de l’Ouest, Nantes, France; 6 Department of Hematology, IUC, Toulouse, France; 7 Department of Hematology, CHU, Lille, France; 8 Lebow Institute of Myeloma Therapeutics and Jerome Lipper Multiple Myeloma Center, Dana-Farber Cancer Institute, Harvard Medical School, Boston, MA 02115 USA; 9 Boston Veterans Administration Healthcare System, West Roxbury, MA 02132 USA

## Abstract

Innovative approaches combining regulatory networks (RN) and genomic data are needed to extract biological information for a better understanding of diseases, such as cancer, by improving the identification of entities and thereby leading to potential new therapeutic avenues. In this study, we confronted an automatically generated RN with gene expression profiles (GEP) from a cohort of multiple myeloma (MM) patients and normal individuals using global reasoning on the RN causality to identify key-nodes. We modeled each patient by his or her GEP, the RN and the possible automatically detected repairs needed to establish a coherent flow of the information that explains the logic of the GEP. These repairs could represent cancer mutations leading to GEP variability. With this reasoning, unmeasured protein states can be inferred, and we can simulate the impact of a protein perturbation on the RN behavior to identify therapeutic targets. We showed that JUN/FOS and FOXM1 activities are altered in almost all MM patients and identified two survival markers for MM patients. Our results suggest that JUN/FOS-activation has a strong impact on the RN in view of the whole GEP, whereas FOXM1-activation could be an interesting way to perturb an MM subgroup identified by our method.

## Introduction

Multiple myeloma (MM) is a neoplasm of plasma cells with an incidence rate of approximatively 5/100,000 in Europe. The median survival of MM patients has improved substantially over the past decade. Owing to the establishment of high-dose therapy followed by autologous stem cell transplantation as a routine procedure, significant improvements in supportive care strategies, and the introduction and widespread use of the immunomodulatory drugs thalidomide and lenalidomide, and the proteasome inhibitor bortezomib. Nevertheless, almost all MM patients ultimately relapse, and new drugs and new combinations for the treatment of MM are warranted. MM is a heterogeneous disease at both the clinical and molecular levels. Recent large scale genomics analysis based on the landscape of copy-number alterations and on whole exome sequencing have revealed the hallmarks of genetic changes in MM such as hyperdiploidy, translocations involving the IgH locus, and mutations in the RAS/MAP and NF-kB pathways and in TP53^1^. These genetic changes as well as gene-expression profiling (GEP) have been widely used in the molecular classification of newly diagnosed patients to define diagnostic entities and identify promising new therapeutic targets^2–7^. However, at present a standard of classification based on subgroups that could be targeted therapeutically is still being debated. Clearly, there is a need for innovative tools to improve the identification of the prognostically relevant entities, clinically and biologically, in newly diagnosed MM patients. It is tempting to use the mutational spectrum based on whole-exome sequencing as a gold standard; however we have previously shown that a large number of exome mutant alleles are not expressed clinically or biologically^8^. In addition, exome sequencing may miss potential driver mutations in the non coding regulatory elements known to affect enhancer activity, which thereby affect the transcriptional program^9^; therefore GEP remains a tool of choice. However, GEP alone is limited and must be integrated with innovative approaches that use biological regulatory networks to extract biological information relative to gene expression datasets to provide significant clues about the etiology of myeloma.

During the past decade, many methods of so-called *pathway analysis* or *active pathways detection* have been developed. These methods use as a knowledge base a biological pathway or regulatory network, that compiles a series of molecular phenomena that lead to activation (or inhibition) of gene expression, a cell product such as a hormone, or a physical modification of the cell. Regulatory network information is currently available through databases such as Gene Ontology (GO)^10^, the Kyoto Encyclopedia of Genes and Genomes (KEGG)^11^, the Pathway Interaction Database (PID)^12^, Wikipathway^13^, Transfac^14^, and Causal Biological Networks (CBN)^15^. The main objective of pathway analysis methods is to confront or integrate GEP data with regulatory networks or pathways to distinguish two or more classes of cells (e.g. healthy vs ill) from GEP data by inferring a specific signature for each class. We can identify three principal categories of approaches that have been used to associate GEP with specific pathways^16^.

The Over-Representation Analysis (ORA) group of approaches^17, 18^ includes approaches that are based on differentially expressed (DE) genes. These approaches score single pathways based on the proportion of DE genes (identified with statistical tests or with a threshold) contained in each pathway. In most cases, these methods use a hyper-geometric test^17^ to score each pathway. Moreover, the majority of ORA approaches that use functional annotation (GO) or pathway maps (KEGG) consider the consequences of the DE genes (leading to the differentially expression of proteins) in the associations between gene and pathway. Martin *et al*.^19^ called this type of reasoning *forward assumption* compared to the *backward assumption* ^18^, which considers the causes of those DE genes in the gene-pathway association.

The Functional Class Scoring (FCS) group of approaches uses the full datasets without any pre-selection, allowing integration of the effects of low gene expression variations in the identification of the pathways involved. FCS approaches can use forward^20, 21^ or backward^22, 23^ reasoning. Although these methods improve the problem of genes selection, the pathways in which individual genes are involved are still studied independently. Moreover, the position of the genes in the topology is not used in the analysis.

The Pathway Topology (PT) approaches are very similar to the FCS approaches, but in addition, they score genes according to the pathways to which they belong. Whereas some of these approaches only include interactions between genes^24–27^, others consider different types of relationships between genes^19, 28^, generally activation and inhibition. The majority of methods study each pathway independently. Within this group, we can also identify methods that use both forward^24–27^ and backward^19, 28^ reasoning.

In this work, we propose to integrate the GEPs obtained from myeloma cells (MC) of 602 MM patients and from normal plasma cells (NPC) of 9 healthy donors with the whole compendium of the PID-NCI public pathway repository so as to better understand the mechanisms of plasma cell carcinogenesis. To integrate this data, we first automatically build a directed (and labeled) graph using the whole compendium of the PID-NCI public pathway repository. This graph connects signaling pathways to the transcription of the genes in the GEP dataset. We then integrate the graph with the expression data by reasoning on its logic using IGGY^29^, a tool based on logic programming (Answer Set Programming) that confronts a node coloring (GEP) with labeled and directed graphs. Our combined approach could be considered to fall within the PT category since it takes into account the causality and activation/inhibition logic of graph edges. However, unlike previous methods, it uses a global logic to analyze experimental and pathway data. In this formalism, both forward and backward modes are included as reasoning modes (causes-consequences). IGGY allows us to check the consistency of the information and to generate predictions based upon automatic repairs for upstream non-measured species. It uses DE data as well as the identically expressed genes across classes (invariant genes) in its analysis. The proposed method does not correlate protein activation with gene expression; the two entities are identified separately in the graph. The non-measured protein activations necessary to satisfy the GEP according to the entire pathway database topology are used later to propose a signature for each dataset profile. This global signature can be used to characterize the dataset classes. Moreover, our model also allows us to *in silico* quantify the effect of perturbations on this global pathway for each single patient. We show how this type of method, which combines large-scale information in terms of number of patients, the complete GEP, and the entire compendium database, can be applied to identify new specificities of MM disease compared to normal cells. As a result, we inferred information on the states of specific proteins in the cell that may cause these disorders, and we identified specific markers of MC compared to NPC that can be used to identify survival markers. Furthermore, these markers can be studied as therapeutic targets because of their over-representation and their impact on the involved pathways.

## Materials and Methods

### Data

#### Experimental Procedures

Plasma cells were isolated from the bone marrow of 602 newly diagnosed cases of MM. The samples were obtained during standard diagnostic procedures conducted at the Intergroupe Francophone du Myélome (IFM) centers. The subjects included patients younger than 65 years of age who were enrolled in either the IFM 2005–01 trial (n = 311) or the IFM-2007-02 (n = 128) trial, older patients enrolled in the IFM-2007-01/Multiple Myeloma 020 trial (n = 76) and 9 normal donors. The experiments were undertaken with the understanding and written informed consent of each subject. Plasma cell purification was performed as previously described^30^. Purified plasma cells were frozen at *−*80 °C in lysis buffer. Approval for this study was obtained from the University Hospital of Nantes. The study fulfilled the requirements of the Declaration of Helsinki.

#### Gene expression profiling

RNA was extracted using the AllPrep DNA/RNA MiniKit or the RNeasy Micro kit (QIAGEN, Valencia, CA, USA) in accordance with the manufacturer’s instructions. RNA quality and quantity were assessed using Agilent 2100 Bioanalyzer (Agilent, Palo Alto, CA, USA) and a Nanodrop Spectrophotometer (NanoDrop Technologies, DE, USA), respectively. MM samples from which 50 ng of total RNA was available were processed according to manufacturer’s instructions (NuGEN, San Carlos, CA, USA) before labeling and hybridization onto an Affymetrix Human Exon1.0 chip according to the manufacturer’s instructions (Affymetrix, Santa Clara, CA). Data were analyzed and *log*_2_ normalized with Expression Console Affymetrix software v1.1 using an RMA algorithm.

#### Data discretization

Since our *graph coloring model* requires invariant genes, classical discretization methods cannot be used in our study. To identify over-/underexpressed and invariant gene expression for each profile, we used two thresholds: *k*_1_ for invariant genes and *k*_2_ for variant genes. For each gene *g*, we computed a vector, *p* ^*g*^, composed of its differential expression, ${p}_{i}^{g}$, in each dataset expression profile *i*. A profile *i* refers to each of the 611 cells of type MC or NPC considered in this study. ${p}_{i}^{g}$ values were computed by subtracting the mean expression of g in the NPC set from its gene expression level in MC (Supplementary Material, Figure S1). We then discretized the values of ${p}_{i}^{g}$ using two thresholds *k*_1_ and *k*_2_.

If ${p}_{i}^{g} > {k}_{2}$, g was considered over-expressed for *i*;

if ${p}_{i}^{g} < -{k}_{2}$, g was considered under-expressed for *i*;

and if $-{k}_{1} < {p}_{i}^{g} < {k}_{1}$, g was considered invariant for *i*.

By choosing different combinations of values for *k*_1_ and *k*_2_ (see Supplementary Material), we obtained 150 sets of vectors that contain the discrete overexpressed (+), underexpressed (−), or invariant (0) values for all the genes expressed in each dataset. We discarded combinations of values leading to *p* ^*g*^ vectors with a sign proportion greater than 50%. Each *k*_1_ − *k*_2_ combination was used to test the precision of our approach. We did this 100 times by using 50% of the discretized {+, −, 0} genes’ expression to predict the other 50% for each MC dataset, after which we comparing the measured and predicted data using a precision matrix (Supplementary Material, Table S1). The thresholds leading to the best precision of 43% (IC 95%: ±3%) were *k*_1_ = 0.03 and *k*_2_ = 0.2; these thresholds were used in the remainder of the study to select the variant and invariant genes. In the Supplementary Material, Figure S2, we show the precision obtained for all of the threshold combinations that were selected. Since our discretization method fixes the same thresholds for all genes across all profiles, we also used K-means to discover gene-specific thresholds. However, the precision of K-means methods (for *k* = 3) was lower than that obtained using the selected thresholds (see Supplementary Material, Figure S3). In the same way, to demonstrate the interest of using invariant genes, we computed the precision of recovering 50% of the data using a two-signs model that receives input data and predicts only over- and underexpressed values. The computed precision was 48%. Note that the two-signs model has a precision closer to a random precision distribution (50%), whereas the precision obtained using a three-signs model is farther from the random precision (33%).

### Graph generation

We used the 2012 version of the complete pathways database PID-NCI (Pathway Interaction Database)^12^ and downloaded it in PID-XML format. This database is specialized to include regulatory pathways involved in cancer. The complete graph contains 17,932 nodes (proteins, complexes, genes, transcription or protein modification events) and 27,976 edges (activation or inhibition). To orient our analysis to the expression profiles and to the biological problem at hand, we built a subgraph with signed edges by extracting the downstream events from three signaling pathways (IL6/IL6-R, IGF1/IGF1-R and CD40), all of which are known to include cellular receptors involved in MM^31^, to the over- and underexpressed variant genes from all datasets by the shortest paths. This cycled, directed subgraph was then filtered by deleting all nodes that are not observed and with one predecessor or one successor^32^. This filtering step involves no loss of information with respect to the graph coloring model and allowed us to reduce the complexity of the analysis while maintaining the dependencies between the nodes.

### Sign consistency modeling framework

**Figure 1:**
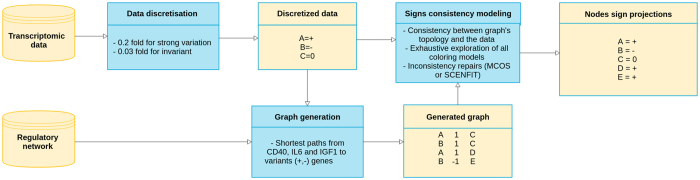
Overview of the sign consistency modeling framework.

In Fig. 1, we illustrate the input (network and transcriptomic data) and output (sign projections) information obtained when the sign consistency modeling is applied. In the following sections, we describe in detail the main modeling steps of this framework.

#### Graph coloring model

Assuming a directed graph G(V, E, *α*) in which V is the set of nodes, E is the set of edges and *α* is a function labeling the edges as *α*:*E* → {+, −}, let *β* be a set of observed data with *β*:*V* → {+, −, 0}. In our case, *β* is obtained from GEP and labels only the nodes that are preceded by a “transcription” event as reported by the PID-NCI database. Thus, there are nodes in V, such as proteins or complexes, that remain unlabeled. Our reasoning framework expresses that there is at least one state or *coloring model* of this biological system. A coloring model is an assignment *μ*:*V* → {+, −, 0} of each node in V to a sign in {+, −, 0}. Let us denote by S the set of all possible coloring models; note that |*S*| = 3^|*V*|^. When imposing the restrictions of *β* to S, we reduce the size of all possible coloring models (${{\rm{S}}}^{\bigstar}$) to 3^|*V*|−|*β*|^.

#### Sign consistency

The sign consistency imposes a reasoning mode over a graph *G* and a labeling *β* (Supplementary Material, Figure S4). This reasoning imposes that each {+, −} variation (in a given coloring model) associated with a node *n* in *V* is explained by the variations in the direct predecessors of *n* in *G*. This notion can be implemented with the following consistency rules, all of which can be verified automatically:

1. All the nodes fixed to be *inputs* are consistent. Usually, these nodes have no predecessors.
2. Each {+, −} variation associated with a node *n* in a given coloring model has to be explained by a direct predecessor of n. That is, each variant node associated with a sign in {+, −} that is not an input needs at least one activator (inhibitor) with the same (opposite) sign.
3. Each invariant node *m* (associated with sign 0) has to be explained either by the fact that (i) all direct predecessors of *m* are associated with an invariant sign, or (ii) at least two direct predecessors of *m* are associated with opposite variant signs {+, −}.

When a graph G is consistent with *β*, then a set $\bar{S}\subseteq {S}^{\bigstar}$ of consistent coloring models can be built. A consistent solution in $\bar{S}$ will be a coloring model in which all the nodes of the graph are colored with respect to *β* and respect the consistency rules.

#### Repairs

When a consistent solution does not exist, the graph topology of *G* is not able to explain the labeling *β* according to the three previously explained consistency rules. In this study, we used two approaches to restore the consistency.

**MCOS-repair:** This repair mode corrects the graph topology. Considering that the graph is not complete (missing information, generation method, etc.), we can suppose that some inconsistencies are caused by events that are missing from the graph. It is possible to correct the graph by adding a set of artificial influences (Supplementary Material, Figure S5). In this case, we use the *cardinal minimal correction set* (MCOS) of artificial influences that can be added to restore the consistency. The MCOS is in general not unique.

**SCENFIT-repair:** This repair mode corrects *β* by considering wrong information in the observed data. *β* will be corrected by switching the sign of the observed nodes so as to minimize the number of switches. The switch of an observed node is quantified by a cost, as described below. The set of possible minimal SCENFIT-repairs is not unique.

1. Changing a variant sign (+, −) to the opposite variant sign will have a cost of 2.
2. Changing an invariant (respectively variant) into a variant (respectively invariant) sign will have a cost of 1.

#### Sign projection

After applying a repair operation, the set of consistent coloring models will be the *union* of the consistent coloring models under each minimal repair. Usually, the number of consistent coloring models is very large, and we use a projection of these models to deduce and propose insights from the graph-observations confrontation. We distinguish 7 sign projections classes:

1. 3 classes are *strong*, meaning that the node has the same sign in all consistent solutions {+, −, 0}.

2. 3 classes are *weak*, meaning that the node has 2 signs in the consistent solutions: *Not*+ (−, 0), *Not−* (+, 0), *change* (+, −).

3. The last class means that a node has three signs in the consistent solutions:? (+, −, 0).

### Key nodes identification

In this analysis, we used the sign projections computed after restoring the consistency using the MCOS-repairs. To compare predictions across individuals using statistical and machine-learning approaches, we decomposed each sign projection result over a node *i* in *V* into a triplet of boolean values. The boolean value expresses whether the couple (*i*, *s*), where s ∈ {+, −, 0}, belongs to the sign-projection result. Since we only focused on sign-projections, the nodes observed in *β* were not considered. To reduce the number of variables, we excluded the boolean value that refers to invariant couples (nodes coupled with “0”). In this way, we represent the sign-projections obtained for each GEP as a boolean matrix *M* of size 2 × *m* × (*N* ^*MC*^ + *N* ^*NPC*^), where m represents the number of nodes in G that were never observed in any GEP and *N* ^*MC*^ (respectively *N* ^*NPC*^) represents the number of profiles in class MC (respectively *NPC*). *M*_*ij*_ stands for the decomposed prediction of node *i* under profile *j*; note that *M*_*ij*_ can be separated into ${M}_{ij}^{+}$ and ${M}_{ij}^{-}$, where ${M}_{ij}^{s}=1$ expresses that node *i* is predicted to be of sign *s* in profile *j*. To identify specific markers of MM, we analyzed *M* and looked for overrepresented values when comparing the vectors belonging to MC with those belonging to NPC. For this, we used two approaches, a machine-learning approach based on supervised learning and a statistical approach based on frequency classification. For the supervised learning, we used a decision tree^33^ and a random forest classification^34^. Due to the underrepresentation of the NPC, we increased the weight of each NPC by 67 so as to have the same order of population in each group (9 NPC and 602 MC). For the frequency approach, we calculated the frequency score (FS) for each group (MC or NPC) and for each assignment (*i*, *s*) as follows:

1 $$F{S}_{i,s}^{C}=\frac{1}{{N}^{C}}\sum _{j=1}^{{N}^{C}}{M}_{ij}^{s},$$

where *C* represents the class MC or NPC and *s* represents the {+, −} sign assigned to *i*. We then sorted our results based on a Fisher test between the proportions for NPC and MC to determine the most specific node assignments for the MC datasets.

### Nodes perturbation

In this analysis, we quantified the effectiveness of a node perturbation (Supplementary Material, Figure S6) to simulate *in silico* the activation or inhibition of a protein. The quantification of these *in silico* perturbations was performed in two steps. First, we considered the set of assignments in *M* (see previous section), where ${M}_{ij}^{s}=1$ expresses that node *i* is predicted to be of sign s in profile j, with s ∈ {+, −}. For each assignment ${M}_{ij}^{s}$, we generated a new dataset of observations ${\beta }_{ij}^{s}$ identical to the original dataset of profile *j* (*β*_*j*_) except that we added an observation on node *i* fixed to *s* ∈ {+, −}. We then computed the SCENFIT score $S{F}_{ij}^{s}$ between the graph *G* and ${\beta }_{ij}^{s}$. The second step consisted of computing the Top Perturbation Score (TPS) for each assignment (*i*, *s*) according to its $S{F}_{ij}^{s}$ across all GEP *j*, as follows:

$$TP{S}_{i,s}^{C}=\frac{1}{{N}^{C}}\sum _{j=1}^{{N}^{C}}\,f(i,s,j),$$

where

$$f(i,s,j)=(\begin{array}{ll}\mathrm{1,} & {\rm{if}}\,S{F}_{ij}^{s}\ge top(S{F}_{kj}^{s}),\forall k\in V\backslash Dom({\beta }_{ij}^{s})\mathrm{.}\\ \mathrm{0,} & {\rm{otherwise}}\mathrm{.}\end{array}$$

In these equations, *C* represents the class MC orv NPC, and *s* represents the {+, −} sign assigned to *i*. The function $top(S{F}_{kj}^{s})$ will compute the threshold score that separates the 10% top-ranked SCENFIT scores of profile *j*, that is, those perturbations that generate the highest number of SCENFIT repairs.

### Software and tools

For the sign consistency analysis, we used IGGY^29^, which makes use of an ASP^35^ description of the consistency problem. The graph generation and the mapping of predictions to the couples node-sign were implemented with Python 2.7 using the package NetworkX^36^. The learning and statistical analysis was conducted using R^37^. The computation associated with testing the consistency of the 611 GEP required 5 minutes on a standard machine. All the calculations of nodes perturbations were conducted using the BIRD infrastructure (www.pf-bird.univ-nantes.fr) with 320 nodes and 1.3To RAM.

### Graphs availability

**Figure 2:**
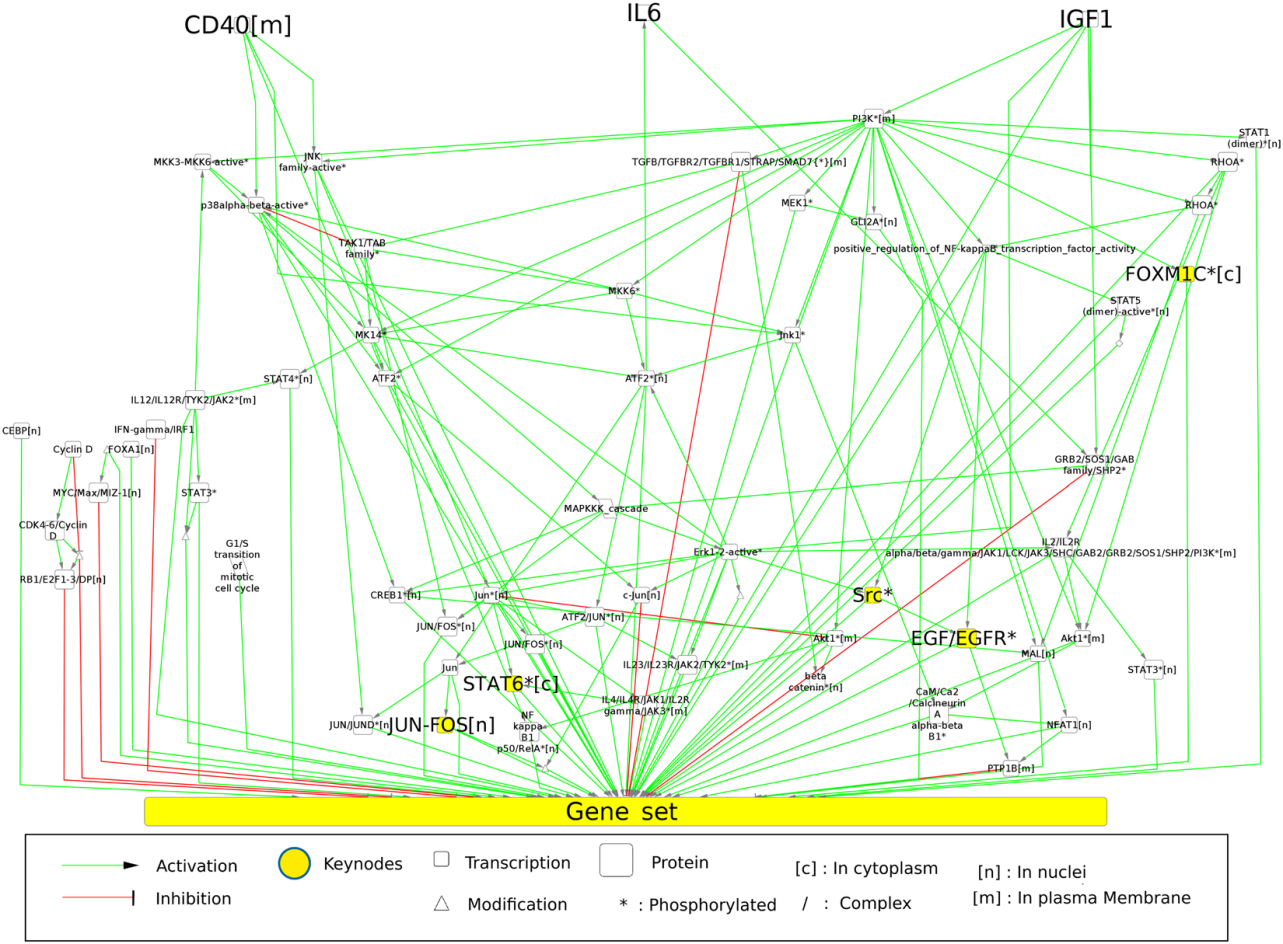
Representation of the subgraph obtained from the PID-NCI database. CD40, IL6 and IGF1 (the nodes in the top portion of the graph) are the 3 queried pathways. The 529 genes that are differentially expressed across all profiles are merged for this representation in the node “Gene set”. The 5 top-ranked nodes according to their FS are labeled in bold type and colored in yellow. We used the same syntax for all nodes in this study. The edges from the “Gene set” node to proteins have been deleted for the sake of clarity.

All graphs used in this study are available online using cynetshare. The subgraphs of NCI-PID before (goo.gl/upfzwC) and after compaction (goo.gl/SfNSv4). The subgraph from Fig. 2 is available at goo.gl/YgHvtQ. The cytoscape session containing all graphs is available at goo.gl/V1Rno5.

## Results

### Data discretization and graph generation

The NCI-PID integration allowed us to find 634 genes (a protein preceded by a transcription event). Independently, our discretization method (Supplementary Material, Figure S1) proposed observations {+, −, 0} on microarray probes corresponding to 15,418 proteins identified in Uniprot. Merging both lists allowed us to identify 557 genes present in the NCI-PID and observed as over/underexpressed or invariant in our GEPs. Variant and invariant genes are distributed across the MC and NPC datasets (Table 1). By extracting the downstream events from three signaling pathways (IL6/IL6-R, IGF1/IGF1-R and CD40)^31^ to the variant genes, we generated an induced subgraph from NCI-PID containing 2,269 nodes, 2,683 edges and connecting 529 variant genes. This graph was then compacted to a new graph with 596 nodes and 960 edges (Fig. 2) and composed of 529 observed nodes (genes) and 67 unobserved nodes, including 23 proteins, 33 complexes, 2 biological processes, 9 proteins reactions (translocation, phosphorylation, etc.).

**Table 1:** Observed and predicted data repartition between NPC and MC.

Signs	Observed data		Predicted data	
	NPC	MC	NPC	MC
+	34%	38%	30%	31%
−	34%	51%	29%	36%
0	32%	11%	14%	3%
change	—	—	7%	6%
Not+	—	—	2%	1%
Not−	—	—	3%	1%
?	—	—	15%	22%
Total	2085	210975	3279	153181

Observed data are the data extracted from the gene expression profiles. Predicted data are the sign projections predicted after the confrontation between the observed data and the PID-NCI graph. In the last row, we show the total observations and predictions across all profiles.

### Validation of predictions

**Figure 3:**
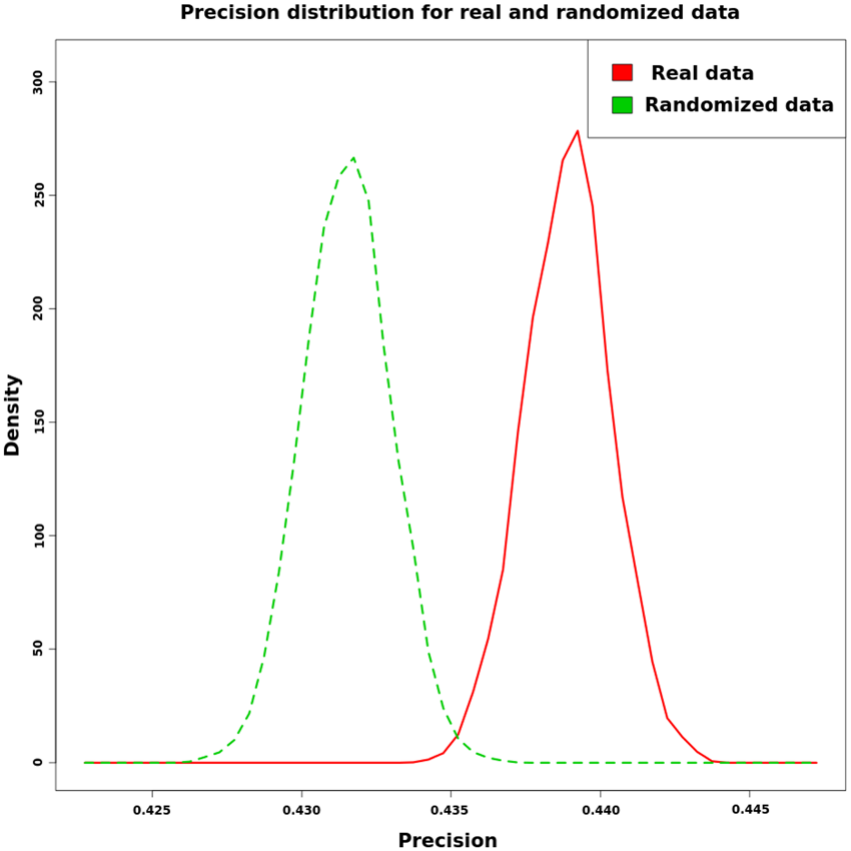
Precision distribution of our method with real observed and randomized data.

The confrontation between the data and the graph topology allowed us to predict the node signs for each dataset (Table 1). To validate our predictions, we compared the precision of the predictions with that of the randomized data. In this case, we used 50% of the measured genes {+, −, 0} to predict the other half of the genes for each sample; we performed the same process after randomizing the data and repeated this computation up to 1000 times. We obtained two sets of precisions (Fig. 3; Supplementary Material, Table S1). A two-tailed t-test yielded a p-value lower than 2.2e–16. This shows the efficiency of our prediction method in comparison with random precision.

### Identification of specific node assignments for MC

**Figure 4:**
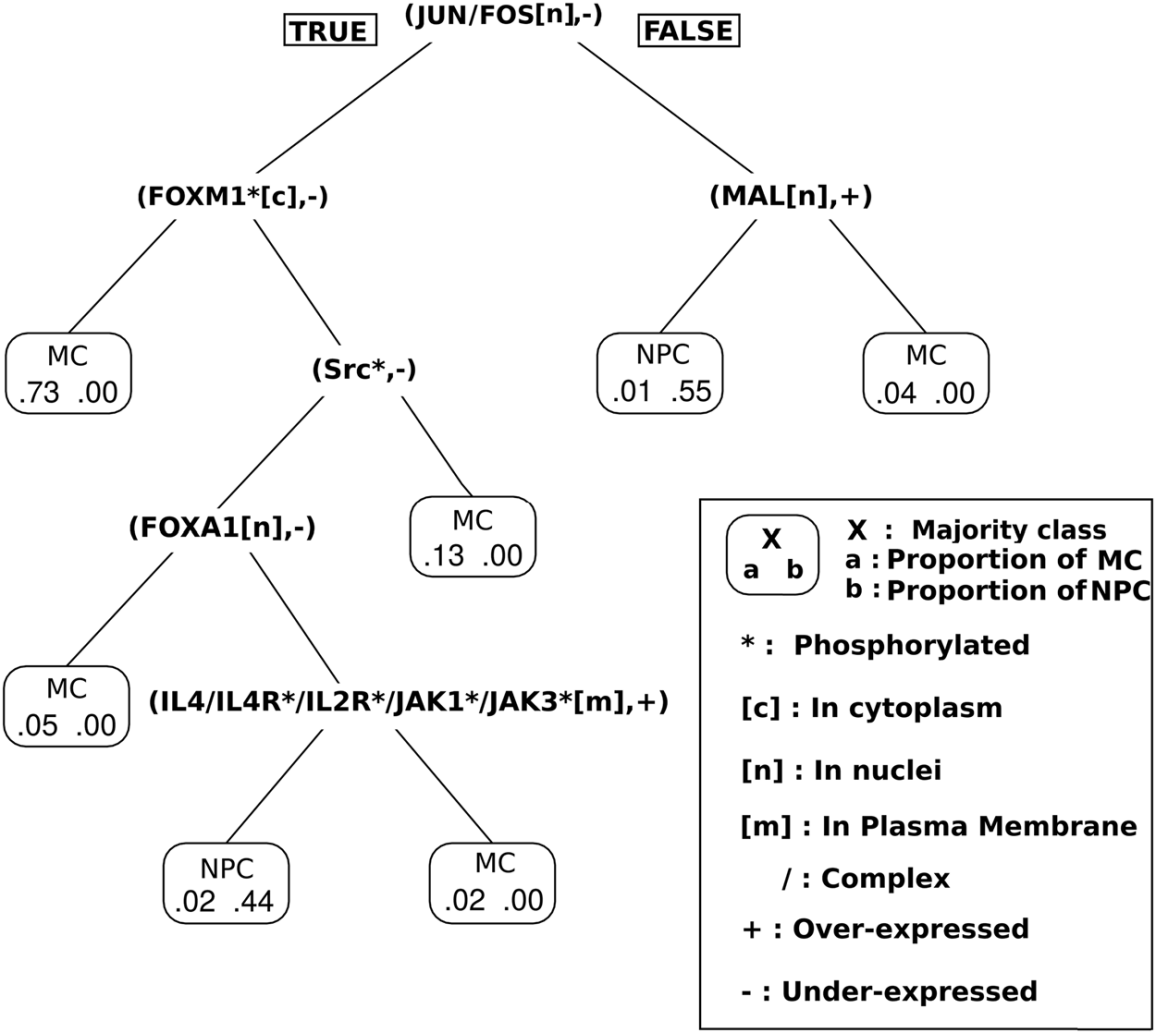
Decision tree based on predicted node-sign assignments.

To identify MC subgroups, we applied a decision tree algorithm to the presence/absence value of a sign prediction (see Methods section). This result is illustrated in Fig. 4. It shows that the combination of the assignments (JUN/FOS[n], −) and (FOXM1*[c], −) is associated with the majority of MC (73%) and that the method can distinguish MC from NPC. JUN/FOS[n] represents the protein complex composed of JUN and FOS, which is located in the nucleus, whereas FOXM1*[c] represents the FOXM1 protein, which is phosphorylated and located in the cytoplasm. The full node syntax is given in Fig. 2. Moreover, we can identify another important group of MC (13%) that is characterized by the presence of (JUN/FOS[n], −) and the absence of (FOXM1*[c], −) and (SRC*, −). Similar results were obtained using a random forest classification (Supplementary Material, Figure S7).

To characterize the shared specificity for all MC, we computed the frequency scores (FS) for our predictions (see Methods section). The complete list of the FS obtained is shown in Table S2 of the Supplementary Material. In Table 2, we show the 5 best p-values associated with a Fisher test with *FS*_*MC*_ > *FS*_*NPC*_. For these assignments, we checked the number of input/output variant genes connected to each node in the graph (Table 2, column connectivity). We observe that inhibition of the complex JUN/FOS[n] is predicted for 95.6% of MC. The activity levels of FOXM1*[c] and STAT6*[c] were predicted to decrease. This decrease, in terms of protein activity, is correlated with the level of gene expression in 76% and 93%, respectively, of the MC datasets. The FS classification identified the presence of (Src*, +) as an interesting marker for MC datasets. Interestingly, the decision tree approach identified the absence of (Src*, −) as distinguish MC datasets that were previously characterized by (JUN/FOS[n], −) and (FOXM1*[c], −). Both the machine-learning and statistical methods identified (JUN/FOS[n], −) and (FOXM1*[c], −) as important markers of MC datasets. In Fig. 2, we show (marked as yellow nodes) how these 5 main proteins or protein complexes appear connected following the PID-NCI representation.

**Table 2:** 5 top-ranked results for the frequency analysis for MC signatures.

Predicted node	Sign	*FS* ^*NPC*^	*FS* ^*MC*^	p.val (Fisher)	References	Connectivity	OVE	
							+	−
JUN/FOS[n]	−	0.444	0.956	2.65E-005	38–42	8/529	373	137
FOXM1*[c]	−	0.222	0.774	7.97E-004	43, 44	529/529	85	265
STAT6*[c]	−	0.222	0.764	1.05E-003	∅	8/529	30	429
EGF/EGFR*[m]	+	0.556	0.935	2.08E-003	45–47	529/529	79	4
Src*	+	0.556	0.935	2.08E-003	48–50	529/529	110	48

*FS* ^*NPC*^ and *FS* ^*MC*^ show the frequency scores for NPC and MC, respectively. The references column lists the publications that agreed with our sign prediction. Connectivity refers to the ratio of genes connected to each predicted node. The OVE (observed variant expression) shows the repartition of variant gene expression using the best precision threshold without considering graph information.

### JUN/FOS activity as specific marker

The FOS and JUN proteins form a heterodimer complex that is responsible for AP-1 activity. This activity is known to play a role in tumorigenesis because it has been implicated in the induction of apoptosis, in the promotion of cell survival and in proliferation. The classification methods showed that (JUN/FOS[n], −) is the best assignment to distinguish MC from NPC and revealed that AP-1 activity is lower in almost all MM patients than in normal controls. Inspection of individual patients’ subgraphs showed predominantly underexpression (65% of the observed expression in MC) of the proapoptotic protein BIM (Supplementary Material, Figure S8). These results are in agreement with the results of *in vitro* studies demonstrating that in myeloma cell lines IL6 protects against apoptosis via AP-1 inactivation^39^.

### FOXM1 activity as survival marker

**Figure 5:**
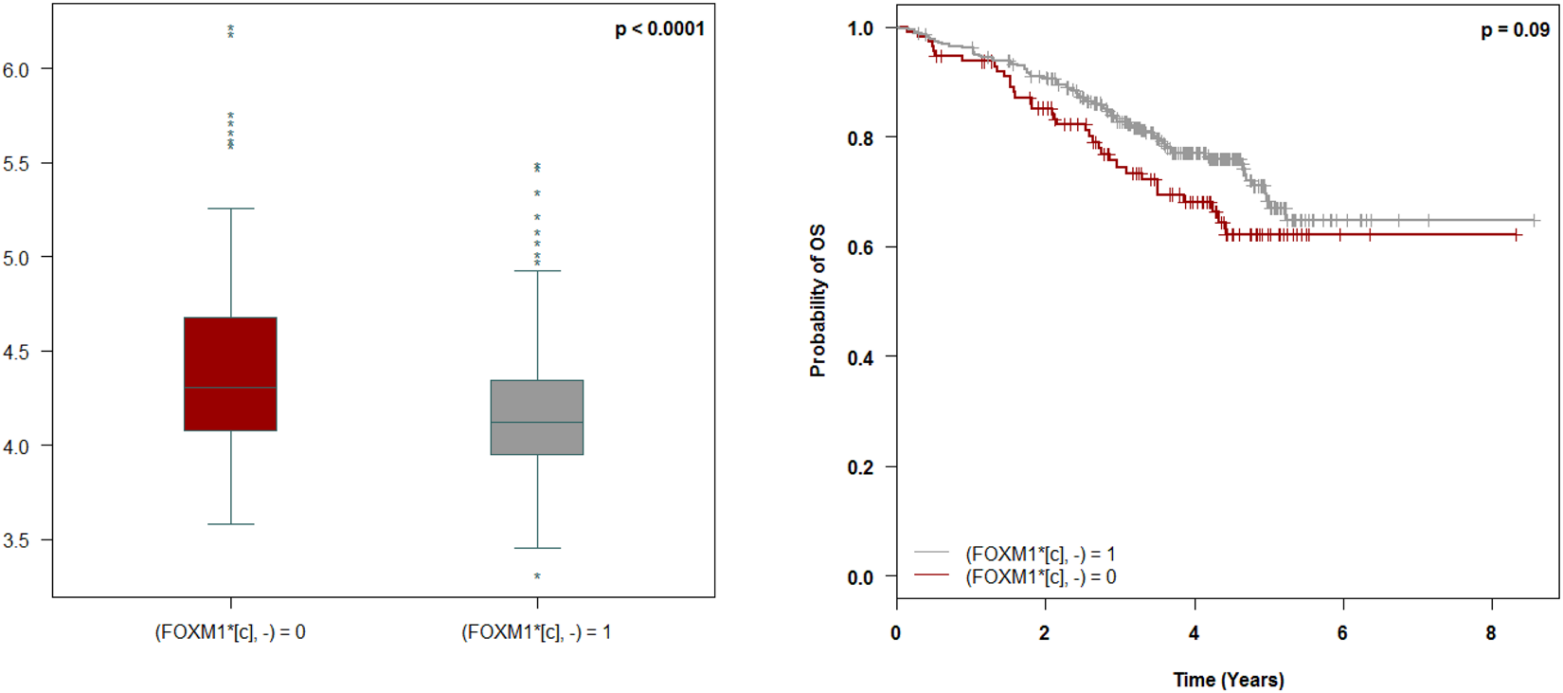
(Left) Gene expression of FOXM1 among MC datasets with or without the prediction (FOXM1*[c], −). (Right) Overall survival (OS) of patients with prediction (FOXM1* [c], −) or without prediction (FOXM1* [c], −).

FOXM1, a transcriptional factor known to be associated with MM, has been studied as a therapeutic target^44^. Based on the graph reduction and on our reasoning model, FOXM1*[c] is equivalent to FOXM1*[n] and is representative of the FOXM1 transcriptional activity. Firstly, we analyzed *FOXM1* gene expression in the MC groups in which FOXM1 activity was predicted. We found that decreased FOXM1 activity is associated with reduced expression of the *FOXM1* gene (Fig. 5, left). Since our model identified a subgroup of patients with decreased activity of FOXM1 and since decreased expression of *FOXM1* is associated with superior survival (Supplementary Material, Figure S9), we wanted to know whether FOXM1 activity could impact survival. We compared overall survival (OS) in both predicted groups in the larger cohort of patients that received comparable treatment (Velcade-dexamethasone induction followed by high-dose melphalan and autologous stem cell transplantation; n = 450) (Fig. 5, right). A log-rank test between these groups yielded a p-value of <0.1, allowing us to conclude that low FOXM1 activity is associated with a trend towards better survival.

### Improvement of the current prognostic model in MM using node variables

Univariate and multivariate Cox proportional hazards analyses were performed on the cohort of 450 MM patients who received comparable treatment to determine the relative prognostic values of the 201 couples combining unobserved nodes and all signs (+, −, 0) and the three strongest known prognostic variables in MM (Table 3); these were the translocation of chromosomes 4 and 14 (t(4;14)), the deletion in the short arm of chromosome 17 (del(17p)) and serum 2-microglobulin ≥5.5 mg/L (*β*_2_-microglobulin) for OS determination^51^. In the multivariate analysis, estimation of hazard ratios for death indicates that both (G1/S transition of mitotic cell cycle, −) and (RB1/E2F1-3/DP[n], +) were independent powerful prognostic factors (Supplementary Material, Figure S10).

**Table 3:** Parameters Associated With Overall Survival.

Parameters	Univariate analysis			Multivariate analysis		
	HR	95%CI	P.value	HR	95%CI	P.value
*β*_2_-microglobulin, mg/L ≥5.5 *v* <5.5	2.03	1.35–3.05	0.001	1.53	0.99–2.35	0.056
t(4,14), Yes *v* no	3.19	2.08–4.89	<0.01	2.41	1.49–3.90	<0.01
del17p > 60 *v* ≤60	4.16	2.53–6.83	<0.01	3.16	1.80–5.56	<0.01
(G1/S transition of mitotic cell cycle, −), yes *v* no	0.33	0.22–0.47	<0.01	0.47	0.30–0.72	<0.01
(RB1/E2F1–3/DP[n], +), yes *v* no	0.49	0.33–0.75	0.001	0.58	0.36–0.93	0.025

The multivariate model with the known prognostic parameters shows that these factors increase the log-likelihood from −515.16 (null model) to −496.62 (3 parameter model), with p-significance <10^−7^ (null model vs 3 parameter model) whereas the parameters (G1/S transition of mitotic cell cycle, −) and (RB1/E2F1-3/DP[n], +) increase the log-likelihood from −496.62 (3-parameter model: AIC3p = 999.2) to −486.90 (5-parameter model: AIC5p = 983.8) with p-significance <10^−4^ (3-parameter model vs 5-parameter model) and <10^−10^ (null model vs 5-parameter model). Therefore, we can conclude that the 5-parameter model provides more prognostic information than the 3-parameter model (AIC5p < AIC3p and p5p vs 3p <10^−4^). In term of the global increase in the log-likelihood between the null model and the 5-parameter model, the specific impact of the selected pairs represents more than 34% of the total.

### Node perturbation

From the computation of all *in silico* node perturbations (see Methods section), we evaluated the impact of perturbing the key nodes found with the FS method (Table 4). A unilateral Fisher test allowed us to evaluate the significance of each perturbation compared to the NPC datasets. We can see that the activation of JUN/FOS generates a top-ranked (10% top) score of conflicts and therefore repairs 74.6% of the MC datasets, whereas it repairs only 22.2% of the NPC datasets. Interestingly, *in vitro* JUN overexpression in MM cell lines results in cell death and growth inhibition^41^. A similar tendency (more conflicts in MC than in NPC) is observed when FOXM1 is activated, but the difference cannot be considered significant. Nonetheless, we note that of the 36.4% of profiles in which the activation of FOXM1 is top-ranked, 96.8% correspond to patient profiles with the prediction (FOXM1*[c], −) (Supplementary Material, Table S3). For the other proteins and complexes, we can see that the difference between MM and NPC is not significant. It is worth noting that the p-value of a perturbation that goes in the opposite direction of the prediction shown in Table 2 is in all cases lower than the one of a perturbation which goes in the same direction of the prediction.

**Table 4:** Top perturbation score for nodes identified with the FS method.

Node	Dir.	*TPS* ^*NPC*^	*TPS* ^*MC*^	p.val
JUN/FOS[n]	+	**22.2%**	**74.6%**	0.001
	−	44.4%	0.5%	1
FOXM1*[c]	+	**11.1%**	**36.4%**	0.107
	−	55.6%	19.1%	0.997
STAT6*[c]	+	**33.3%**	**55.0%**	0.169
	−	44.4%	21.9%	0.970
EGF/EGFR*[m]	+	0.0%	0.3%	0.971
	−	**0.0%**	**3.5%**	0.728
Src*	+	0.0%	1.3%	0.887
	−	**11.1%**	**33.4%**	0.150

Dir stands for the direction of the perturbation (+, activation and −, inhibition). TPS represents the frequency with which perturbing a node in a specific direction was significant (i.e. it generated a high, 10% top, SCENFIT score) across the MC profiles (*TPS* ^*MC*^) or NPC profiles (*TPS* ^*NPC*^). The bold percentages refer to perturbations that have a direction opposite to that of the predicted signs obtained with the frequency score (Table 3). P.val was obtained using a unilateral Fisher test.

## Discussion

### Data discretization and graph generation

Our method incorporates both differential and similar expressions in its reasoning. All *pathway analysis* methods reviewed in the Introduction use the difference in gene expression between the two classes of subjects to extract the specific signatures. The similarity of expression between classes is not used in the ORA and FCS approaches because these methods base their analyses on the differential expression of genes. The PT approaches reviewed use only differential gene expression in their reasoning. We believe that adding information on similar expression enables us to better capture cellular behavior. The results of the precision analysis using a two-sign coloring model tend to support this hypothesis. Our method differs from classic pathway analysis methods in that it incorporates the notion of automatic reasoning. Within the context of MM, we are able to automatically detect repairs. These repairs are specific for each GEP and could represent cancer mutations, regulatory network incompleteness or experimental errors. The graph used in this study contains 529 genes; we can therefore observe the strong connectivity that exists among PID pathways since the total number of genes in the PID is 634. This strong connectivity is important for methods such as ours that are able to reason on the information content of the whole database. We observe, however, that the number of genes connected to cancer pathways in PID is far below the total number of human genes. This underrepresentation of regulatory knowledge is an important limitation of PID. Apart from this fact, PID-NCI includes important modeling information that identifies *transcription events*. This information allows us to separate gene expression from protein activity. These two parameters are not necessarily correlated, especially in cases involving phosphorylated proteins or complexes such as JUN/FOS.

### Key nodes identification

Our analysis of the predictions made by the method allowed us to identify nodes associated with a sign specific to MC compared to NPC datasets. Among these assignments, we found the inhibition of JUN/FOS[n] and FOXM1*[c]. These proteins are known to be involved in cancer in general^52, 53^ and in hematological malignancies in particular^43, 44^. In the case of FOXM1, we showed that this transcription factor can represent a survival marker when its activity decreases. We can draw a parallel with the bibliography, which identifies the activation of the FOXM1 pathway as a risk factor. For JUN/FOS, our analysis identified this pathway as a potential therapeutic target but not as a survival marker. We observed that inhibition of the associated pathways has been already identified in MM patients^39, 41^ and in patients with other cancers. Moreover, this pathway is targeted in some therapeutic approaches^38, 40^. We identified two couples that improve classical prognostic models. In the case of the first couple, (G1/S transition of the mitotic cell cycle, −), we can associate this node with the proliferation pathway. The computed prognostic model showed that the prediction of inhibited proliferation can be a protective factor for MM patients. The second node, (RB1/E2F1-3/DP[n], +), was also identified as a protective factor by the 5-parameter model. This complex is known to be involved in the RB pathway, which influences cell growth pathways by regulating the initiation of DNA replication. This pathway is usually altered in cancer, leading to a loss of function^54^, and current therapeutic approaches aim to activate this pathway^55^.

Using the *in silico* node perturbation method, we were able to estimate the effect of perturbing a node within a particular dataset (*i.e*. single patient cancer cell). This method represents a powerful tool for analyzing the consequences of perturbations of oncogenic pathways in a given patient, especially as *in-vitro* experiments are limited due to the small amount of viable myeloma cells that are obtained after bone marrow aspiration. The results of this *in silico* analysis show that activation of JUN/FOS[n] had a significant impact on 75% of MC profiles; all of these JUN/FOS[n] = “+” sensible MC profiles had the prediction (JUN/FOS[n], −). In addition, activating FOXM1*[c] had a significant impact on 36.4% of the profiles; 96.8% of the FOXM1*[c] = “+” sensible MC profiles had the prediction (FOXM1*[c], −). The difference in the percentages of JUN/FOS[n] and FOXM1*[c] can be explained by the graph topology and the connectivity of the individual nodes. JUN/FOS[n] is connected to eight genes through a distance of 1 molecular species (Supplementary Material, Figure S8); therefore, perturbing JUN/FOS[n] will impact these genes directly since they are strongly constrained by the sign of JUN/FOS[n]. On the other hand, FOXM1 is connected to 529 genes through longer paths through distances of from 4 to 77 molecular species. These genes may have other predecessors that are independent of FOXM1; this could explain why activation of FOXM1 has a strong effect on only 37% of the MC profiles. Overall, we think that this *in silico* method could be used to reinforce the choice of a therapeutic target for a specific patient profile.

## Conclusion

In this study, we used a specific approach to study and understand the heterogeneous gene expression profiles of approximately 600 multiple myeloma (MM) patients. Our primary goal was to provide mechanistic scenarios by identifying protein activity states of molecules that may be central to the diversity of gene expression. Our approach relies heavily on reasoning based on graphs and on changes in gene expression in the form of logical programs that combine these two types of information. The method proposed here can be summarized in the following steps. First, we obtained a directed graph, allowing us to connect significantly up-/down-regulated genes to upstream MM-related cellular receptors. Second, we confronted this graph to transcriptomic data with IGGY, which is a tool that reasons on the logic of the graph and on shifts of expression in the data so as to predict (*node*, *sign*) assignments representing the specific states of biological entities. Using two approaches of classification, we were able to identify specific assignments for MC datasets compared to NPC datasets. Finally, taking advantage of our modeling framework, we studied the effect of performing single *in silico* perturbations.

One advantage of this method is that it makes it possible to infer information about protein states from transcriptomic data by using the causal nature of the interactions as documented in PID. This can be interesting when constructing biological models and, more specifically, when developing cancer models for which proteomic data are not always available and extractable, whereas transcriptomic data are easier to obtain. Moreover, compared to the previously presented classical pathway analysis methods, we identify not only the specific biological processes that are implicated in cancer profiles but also the mechanisms associated with those phenomena. After statistically testing the quality of the method’s predictions, we proposed a set of five top-scoring proteins based on their respective changes in activity in MC compared with NPC. We found the AP-1 complex and the FOXM1 transcription factor to be concomitantly inactivated in a strong majority of patients regardless of treatment or age. Interestingly, this method identified a subgroup of MM patients with increased FOXM1 activity associated with poor survival. These findings allow us to validate the predictions of our approach and show that it is feasible to individualize or restrict the analysis of multiple expression profiles to identify markers within subgroups of profiles and to identify parameters associated with survival in these subgroups. The 5-parameter model including the two predicted nodes improves the standard prognostic model in MM. In addition to its strong prognostic value, our model revealed two nodes, (G1/S transition of mitotic cell cycle, −) and (RB1/E2F1-3/DP[n], +), that are of potential biological interest in the understanding of the molecular mechanisms underlying resistance to treatment. Note that these nodes can only be predicted with the graph and coloring model, since they are a (logical) consequence of the GEP. Our results on *in silico* perturbations of a system are also encouraging because they show that changes in the activity of the predicted proteins can serve as input information for conducting efficient perturbations. In this work, we focused only on single perturbations, since they are more experimentally realistic. As a perspective of this work, we wish to deepen the graph vs. gene-expression confrontation analysis so as to understand the differences between MM subgroups based on age, prognosis and other criteria. In this context, one line of research would be to study minimal subsets of perturbations. Another possible line of research would be the classification of gene expression profiles based on plausible graph-coloring models.

## Electronic supplementary material

Supplementary Materials
