# Supplementary material for: Logic programming reveals alteration of key transcription factors in multiple myeloma
Source: Sci Rep. 2017 Aug 23;7:9257. doi: 10.1038/s41598-017-09378-9 (PMC5569101; doi:10.1038/s41598-017-09378-9)
Supplement: Supplementary file 1 — Supplementary Materials [file 41598_2017_9378_MOESM1_ESM.pdf]

Supplementary material: Logic programming reveals alteration of key transcription factors in multiple myeloma

Bertrand Miannay[1,2], Stéphane Minvielle[2,3], Olivier Roux[1], Pierre Drouin[1], Hervé Avet-Loiseau[4], Catherine Guérin-Charbonnel[2,5], Wilfried Gouraud[2,5], Michel Attal[6], Thierry Facon[7], Nikhil C Munshi[8], Philippe Moreau[2,3], Loic Campion[2,5], Florence Magrangeas[2,3,*], and Carito Guziolowski[1,*]

[1]LS2N UMR 6004, École Centrale de Nantes
[2]CRCINA, INSERM, CNRS, Université d'Angers, Université de Nantes, Nantes, France
[3]CHU de Nantes, Nantes, France
[4]Unit for Genomics in Myeloma, IUC-Oncopole; and CRCT INSERM 1037, Toulouse, France
[5]Institut de Cancérologie de l'Ouest, Nantes, France
[6] Department of Hematology, IUC, Toulouse, France
[7]Department of Hematology, CHU, Lille, France
[8]Lebow Institute of Myeloma Therapeutics and Jerome Lipper Multiple Myeloma Center, Dana-Farber Cancer Institute, Harvard Medical School, Boston, MA, 02115, USA Boston Veterans Administration Healthcare System, West Roxbury, MA, 02132, USA
[*]Corresponding authors: Carito.Guziolowski@ls2n.fr and Florence.Magrangeas@univ-nantes.fr

Data discretization method

In order to discretize the measured gene expression in three classes: over-expression (+), under-expression (-), and invariant (0) with respect to the normal plasma cells (NPC) cells, we used a combination of twho thresholds k_1 and k_2. In Fig. 1 we illustrate this selection.

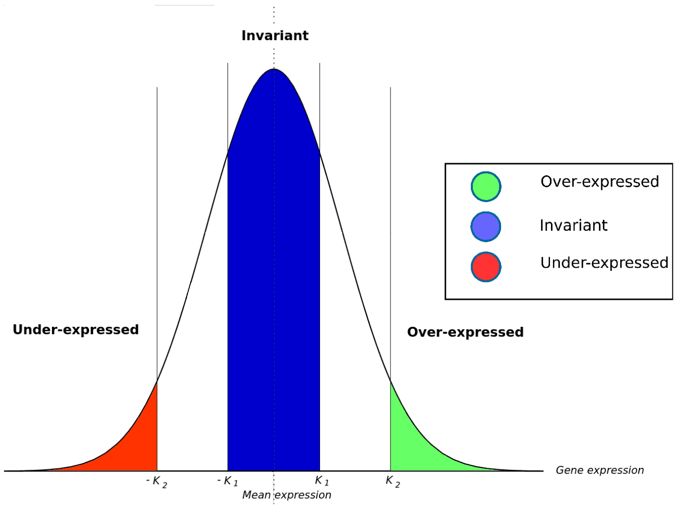

Figure 1: Discretization method based on mean expression with two symetric thresholds

The values selected for k_1 and k_2 were:

- $k_1 \in \{0, 0.01, 0.02, 0.03, 0.04, 0.05, 0.1, 0.15, 0.2, 0.3\}$ for invariant genes.
- $k_2 \in \{0.2, 0.4, 0.6, 0.8, 1, 1.2, 1.4, 1.6, 1.8, 2, 2.2, 2.4, 2.6, 2.8, 3\}$ for over/under-expressed genes.

Computing the Precision of IGGY sign-projection

Once the data was discretized, we selected the best combination of k_1 and k_2 by running IGGY on the network selecting only 50% of the gene expression discretized data, generating sign-projections (predictions), and searching to correctly predict the sign of the other 50% of the data. Since the sign projections outputted by IGGY can be uncertain (weak) values such as CHANGE, Not+, and Not-, we decided to weight the correctness of such weak predictions with a lower (0.5) score than the one used to weight the strong predictions (+, -, 0). In Table 1 we show the weights used to compute the total precision. These weights are 1 for the true strong predictions, 0.5 for the weaks, and 0.3 for the last in order to take into account the informativeness of each class of predictions.

Table 1: Precision matrix used for the computation of precisions.

	Discretized Measured Sign		
Predicted projections	+	-	0
+	1	0	0
-	0	1	0
0	0	0	1
CHANGE	0.5	0.5	0
Not+	0	0.5	0.5
Not-	0.5	0	0.5
?	0.3	0.3	0.3

Precision repartition for each threshold combination

We removed the $k1 - k2$ combinations that leaded to datasets where a gene was assigned in more than 50% of the MC or NPC profiles the same sign. We computed the precision of the prediction of sign-projections with the rest of $k1 - k2$ combinations and the precision results are shown in Fig. 2. We can see that all precisions are situated in the range of 29% to 42%. The combination of thresholds that obtained the best precision was $k_1 = 0.03$ and $k_2 = 0.2$.

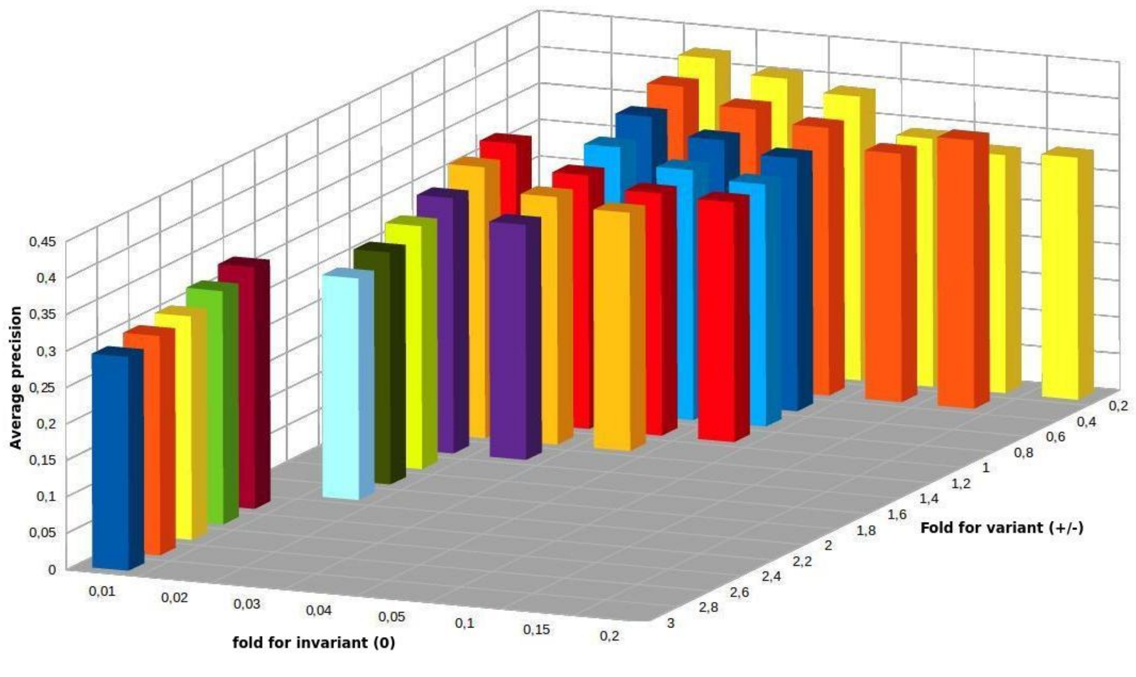

Figure 2: Precision repartition for each threshold combination

Comparison with K-means discretization

To validate our discretization method, we compared the precision obtained with the $k1 - k2$ combination choosed previously with a k-means clustering, with $k = 3$ (-,0,+). These precisions are shown in Fig. 3. A two tailed t.test betweend these two precisions repartitions gave us a p-value lower than 2.2e-16 allowing us to conclude to the efficiency of our discretization approach compared to k-means discretization.

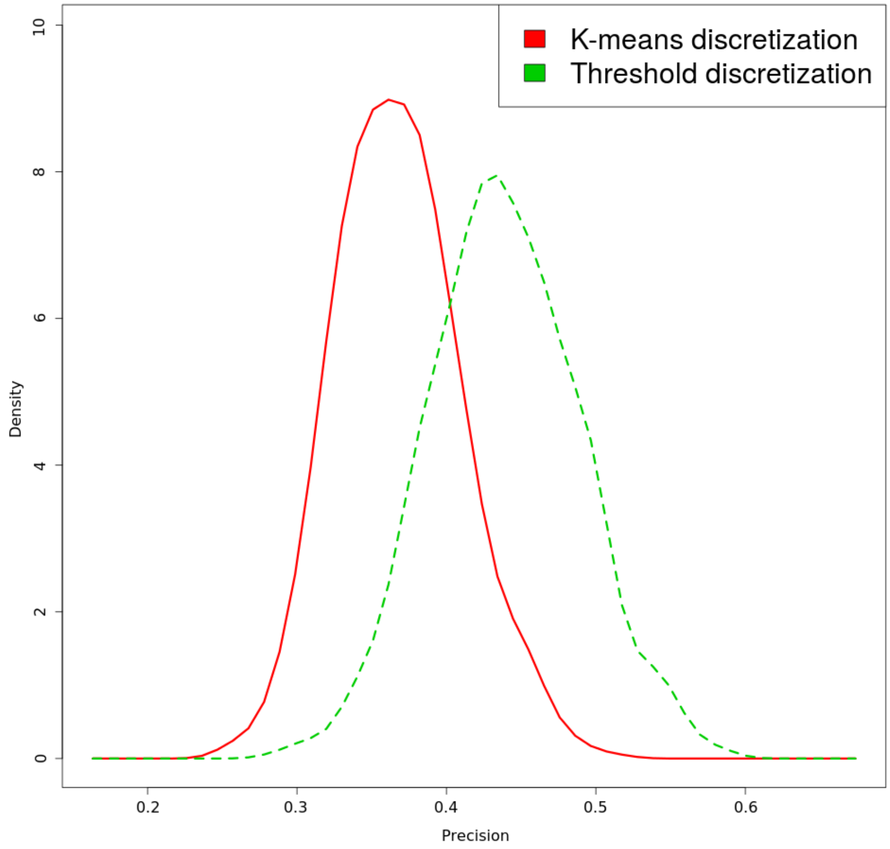

Figure 3: Precision repartition for threshold and k-means discretization

Sign consistency

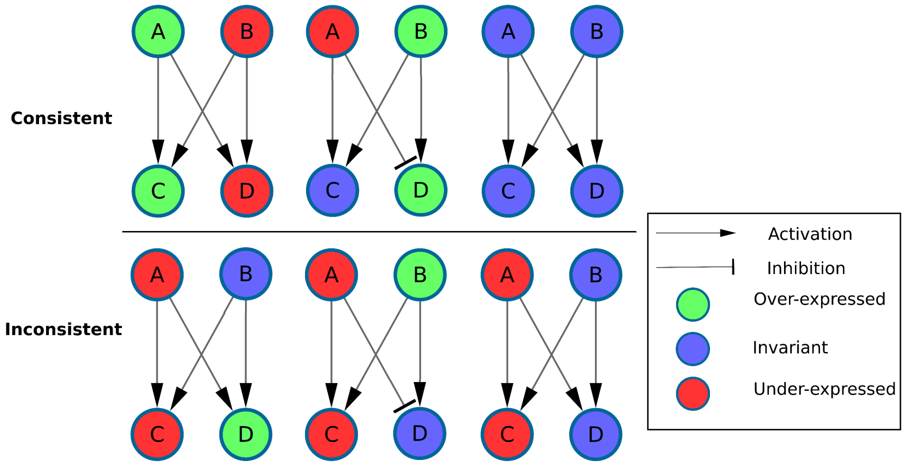

Figure 4: Example of consistent and inconsistent coloring models.

Repairs

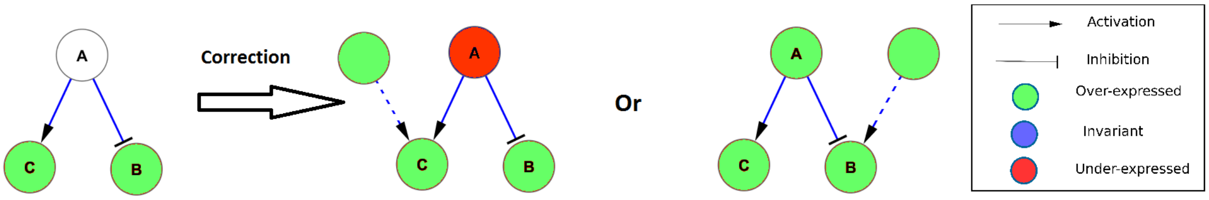

Figure 5: Example of inconsistency correction using 2 MCOS-repairs.

Nodes perturbation

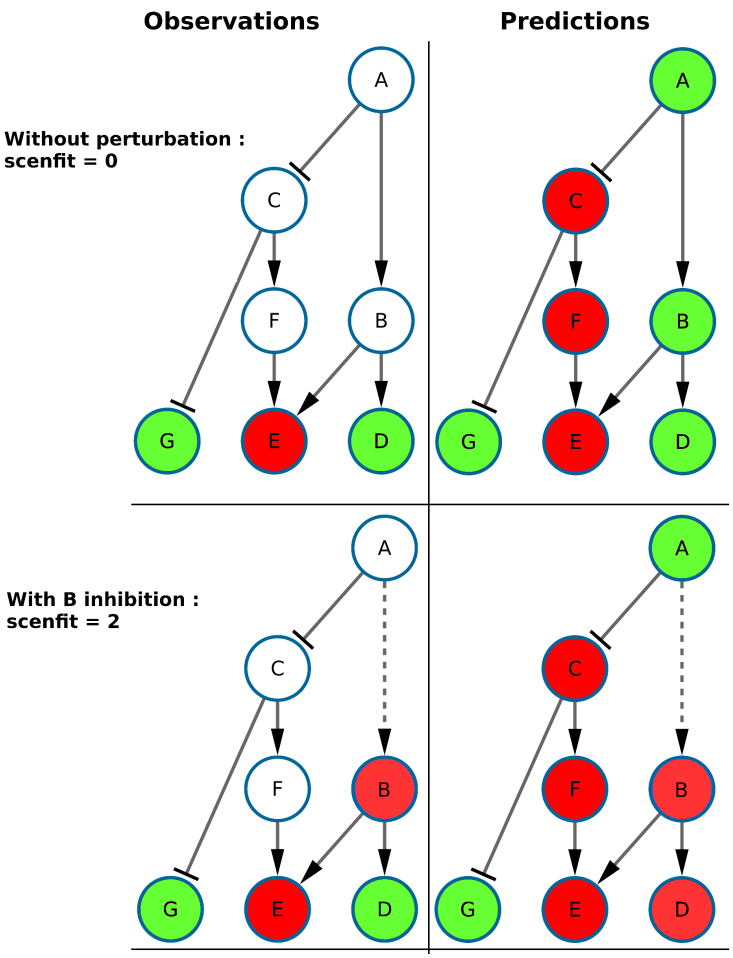

Figure 6: Example of a node perturbation scenario where node B is set to $-$ and this fact generates a SCENFIT score of 2.

Random forest

We show the result obtained with a random forest analysis. The principal couples found by this approch are nearly the same as with the frequency analysis (Figure 7). Nonetheless, this approach identifies the couples, without taking account if they are specific to a group or another. In the case of our frequency analysis, we decided to only select the couples with a better frequency for MPC.

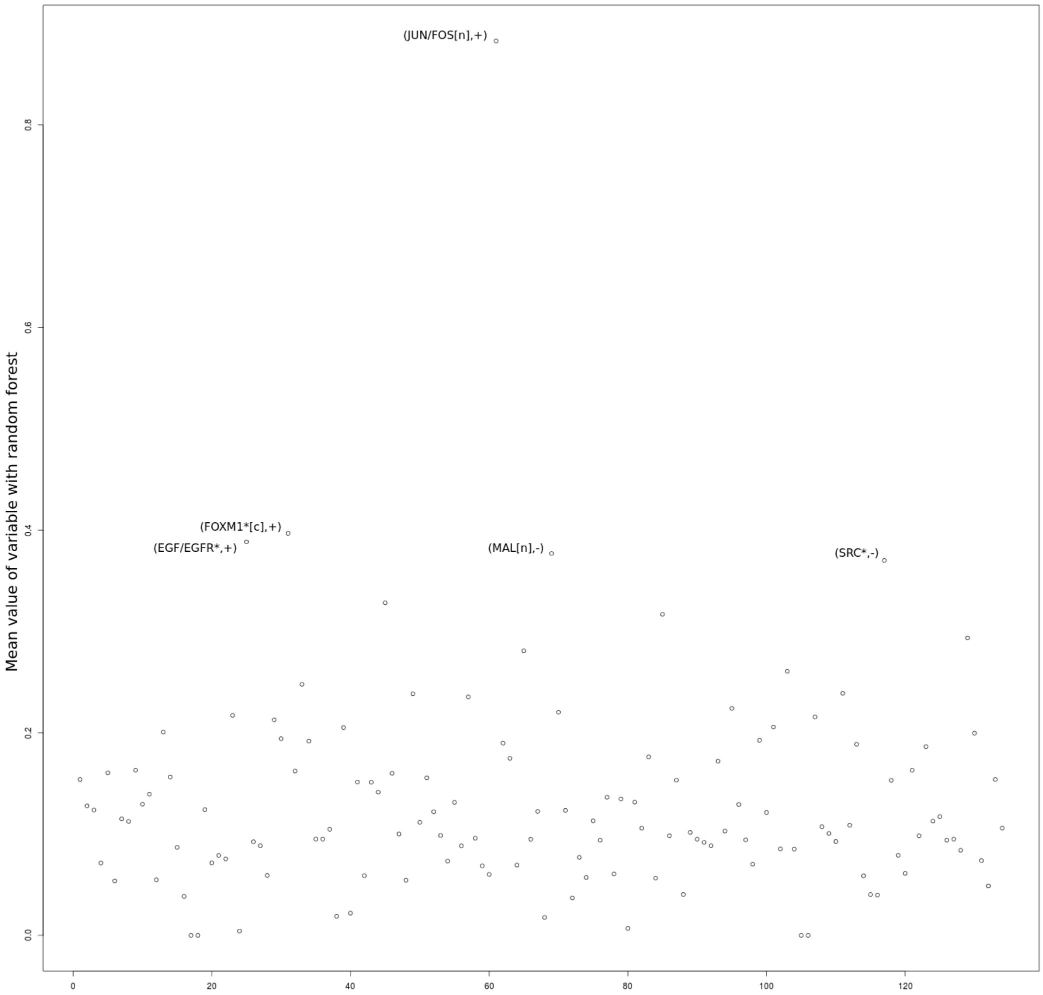

Figure 7: Result for random Forest.

Full list of predictions

In Table 2 we show the prediction of the activity (increase or decrease) of 596 nodes in the graph (proteins, protein-complexes and cellular processes), as well as their frequency score across the 602 MC profiles compared to the NPC profiles. The [m] in a protein name means it is located in the plasma membrane, [n] for the nuclei, and [c] for the cytoplasm. Finally, the asterix in a protein name means that it appears phosphorylated.

Table 2: Results of the frequency analysis

Predicted node	Sign	FS_{NPC}	FS_{MC}	p.value (fisher)
JUN/FOS[n]	-	0,4444444444	0,9568106 312	2,65E-005
FOXM1C*[c]	-	0,2222222222	0,7740863787	0,000796623
STAT6*[c]	-	0,2222222222	0,7641196013	0,0010457727
MAL[n]	+	0,5555555556	0,1013289037	0,0011370116
Src*	+	0,5555555556	0,9352159468	0,0020781468
EGF/EGFR*	+	0,5555555556	0,9352159468	0,0020781468
RB1/E2F1-3/DP[n]	+	0,3333333333	0,7990033223	0,0035098488
NFAT1[n]	-	0,4444444444	0,865448505	0,0039262109
IL23/IL23R/JAK2/TYK2*[m]	-	0,4444444444	0,865448505	0,0039262109
STAT6*[c]	+	0,7777777778	0,3023255814	0,004900412
IL12A/IL12B/IL12R1/IL12R2/TYK2/JAK2*[m]	-	0,5555555556	0,8887043189	0,0134469369
Src*	-	0,7777777778	0,3571428571	0,0134575472
IL23/IL23R/JAK2/TYK2*[m]	+	0,5555555556	0,2026578073	0,0221351528
STAT3*[n]	-	0,3333333333	0,707641196	0,0235380439
G1/S _transition _of _mitotic _cell _cycle	+	0,6666666667	0,2990033223	0,026272743
JUN/FOS*[n]	+	0,5555555556	0,8637873754	0,0263899967
JUN/FOS[n]	+	0,5555555556	0,2176079734	0,0295514303
IL12A/IL12B/IL12R1/IL12R2/TYK2/JAK2*[m]	+	0,5555555556	0,2176079734	0,0295514303
STAT3*	+	0,7777777778	0,3787375415	0,0312675017
IL4/IL4R /JAK1/IL2RG/JAK3	+	0,8888888889	0,5049833887	0,0380656569
MAL[n]	-	0,7777777778	0,9667774086	0,0382477952
positive _regulation _of _NF.kappaB _transcription _factor _activity	-	0,4444444444	0,7607973422	0,0430785009
MKK3-MKK6-active*	-	0,7777777778	0,9634551495	0,0449978118
Jun[n]	+	0,5555555556	0,8388704319	0,0454802151
Akt1*	+	0,8888888889	0,5465116279	0,0466814274
ATF2*[n]	+	0,4444444444	0,7392026578	0,0602829283
ATF2*	+	0,6666666667	0,8970099668	0,0605037172
JNK family-active*	-	0,6666666667	0,8936877076	0,0653292552
IL4/IL4R /JAK1/IL2RG/JAK3	-	0,7777777778	0,9518272425	0,0718166928
CaM/Ca2 /Calcineurin A alpha-beta B1*	-	0,6666666667	0,8887043189	0,0729213452
MK14*	+	0,6666666667	0,8870431894	0,0755452162
PTP1B	-	0,3333333333	0,6445182724	0,0772799188
Akt1*	-	0,5555555556	0,8073089701	0,0794519114
JUN/JUND*[n]	+	0,8888888889	0,5548172757	0,0851744548
FOXA1[n]	-	0,2222222222	0,5548172757	0,0860083332
MKK3-MKK6-active*	+	0,8888888889	0,5813953488	0,08831995
RHOA*	-	0,6666666667	0,8687707641	0,1073656048
G1/S _transition _of _mitotic _cell _cycle	-	0,5555555556	0,780730897	0,1168149651
PI3K*	-	0,5555555556	0,780730897	0,1168149651
CaM/Ca2 /Calcineurin A alpha-beta B1*	+	0,4444444444	0,219269103	0,1168149651
STAT3*	-	0,7777777778	0,9352159468	0,1172093643
Cyclin D	+	0,8888888889	0,9867109635	0,1257988695
JUN/FOS*[n]	+	0,4444444444	0,7059800664	0,1350528502
IFN-gamma/IRF1	+	0,7777777778	0,926910299	0,1423195134
p38alpha-beta-active*	+	0,4444444444	0,6893687708	0,1481743371
ATF2/JUN*[n]	-	0,6666666667	0,8471760797	0,1513916339
JNK family-active*	+	0,5555555556	0,3205980066	0,1579377713
STAT4*[n]	-	0,3333333333	0,6129568106	0,1646594722
TAK1/TAB family*	+	0,7777777778	0,915282392	0,1794778162
MAPKKK _cascade	-	0,6666666667	0,8189368771	0,2176078177
MKK6*	-	0,7777777778	0,9019933555	0,2240161444
Jun*[n]	-	0,5555555556	0,7624584718	0,2294440408
STAT1 (dimer)*[n]	-	0,7777777778	0,8986710963	0,2353951054
NFAT1[n]	+	0,4444444444	0,2508305648	0,241536935
EGF/EGFR*	-	0,5555555556	0,7325581395	0,2612443723
RHOA*	-	0,7777777778	0,8887043189	0,2699151506

Predicted node	Sign	FS_{NPC}	FS_{MC}	p.value (fisher)
IL2R/IL2/JAK1/LCK/JAK3/SHC1/GRB2/SOS1/GAB2/PTN11/PK3CA/P85A*[m]	-	0,5555555556	0,7159468439	0,2854636694
GRB2/SOS1/PTN11*	-	0,4444444444	0,6511627907	0,2902056457
Erk1-2-active*	-	0,4444444444	0,622923588	0,3109884105
positive _regulation _of _NF.kappaB _transcription _factor _activity	+	0,6666666667	0,4568106312	0,3139240743
FOXM1C*[c]	+	0,6666666667	0,4584717608	0,3144645934
PI3K*	+	0,6666666667	0,4617940199	0,315688483
STAT1 (dimer)*[n]	+	0,6666666667	0,4800664452	0,3257703828
ATF2*[n]	-	0,5555555556	0,3936877076	0,329276166
Jnk1*	-	0,8888888889	0,9568106312	0,336027101
MEK1*	+	1	0,8488372093	0,3689753253
IFN-gamma/IRF1	-	1	0,8504983389	0,3695842251
p38alpha-beta-active*	-	1	0,8156146179	0,3758997164
CD40[m]	+	0,6666666667	0,8139534884	0,3811110637
ATF2*	-	0,8888888889	0,73089701	0,4567676076
beta catenin*[n]	+	0,5555555556	0,696013289	0,4666566818
IGF1	+	0,5555555556	0,6910299003	0,4699773265
STAT5 (dimer)-active*[n]	-	0,5555555556	0,6694352159	0,4889925336
CREB1*[n]	-	0,4444444444	0,5996677741	0,4957307553
TGFB/TGFBR2/TGFBR1/STRAP/SMAD7*[m]	+	0,4444444444	0,5897009967	0,4990576611
beta catenin*[n]	-	0,5555555556	0,6561461794	0,5040423859
STAT3*[n]	+	0,3333333333	0,4916943522	0,5054664405
PTP1B	+	0,4444444444	0,5747508306	0,506917695
STAT5 (dimer)-active*[n]	+	0,3333333333	0,4833887043	0,5073910055
Erk1-2-active*	+	0,6666666667	0,5398671096	0,5183589723
Akt1*	+	0,4444444444	0,5564784053	0,520916961
CD40[m]	-	0,8888888889	0,7724252492	0,6917544772
STAT4*[n]	+	0,5555555556	0,634551495	0,7309241973
RHOA*	+	0,6666666667	0,5548172757	0,7378847373
NF kappa B1 p50/RelA*[n]	+	0,6666666667	0,5581395349	0,7379624924
TGFB/TGFBR2/TGFBR1/STRAP/SMAD7*[m]	-	0,5555555556	0,450166113	0,7381429579
MEK1*	-	0,6666666667	0,561461794	0,7381441077
Jnk1*	+	0,6666666667	0,584717608	0,7423231901
Akt1*	-	0,5555555556	0,4817275748	0,7448745865
Jun*[n]	+	0,4444444444	0,3986710963	0,747312076
JUN/FOS*[n]	-	0,4444444444	0,3986710963	0,747312076
GLI2A*[n]	-	0,4444444444	0,5033222591	0,7510723201
MAPKKK _cascade	+	0,7777777778	0,7707641196	1
IGF1	-	0,4444444444	0,4700996678	1
Jun	+	0,4444444444	0,4817275748	1
Jun	-	0,8888888889	0,803986711	1
Jun[n]	-	0,4444444444	0,4534883721	1
CREB1*[n]	+	0,5555555556	0,5581395349	1
MKK6*	+	1	0,9485049834	1
FOXA1[n]	+	0,5555555556	0,5265780731	1
GLI2A*[n]	+	0,6666666667	0,6129568106	1
MK14*	-	0,5555555556	0,5431893688	1
TAK1/TAB family*	-	0,5555555556	0,5913621262	1
JUN/FOS*[n]	-	0,5555555556	0,5448504983	1
MYC/MAX/ZBTB17[n]	+	0,5555555556	0,5913621262	1
MYC/MAX/ZBTB17[n]	-	0,5555555556	0,5531561462	1
IL2R/IL2/JAK1/LCK/JAK3/SHC1/GRB2/SOS1/GAB2/PTN11/PK3CA/P85A*[m]	+	0,7777777778	0,7209302326	1
ATF2/JUN*[n]	+	0,3333333333	0,3089700997	1
RB1/E2F1-3/DP[n]	-	0,4444444444	0,4451827243	1
JUN/JUND*[n]	-	1	0,9551495017	1
CEBP[n]	+	0,4444444444	0,4401993355	1
CEBP[n]	-	0,6666666667	0,6677740864	1
NF kappa B1 p50/RelA*[n]	-	0,6666666667	0,6079734219	1
Cyclin D	-	1	0,9468438538	1
RHOA*	+	0,8888888889	0,8172757475	1
GRB2/SOS1/PTN11*	+	0,8888888889	0,8222591362	1
CDK4-6/Cyclin D	+	1	0,9966777409	1
CDK4-6/Cyclin D	-	1	0,9634551495	1

Example of coloration of JUN/FOS[n] subgraph

In order to predict the sign of each node of the graph, we used the observed data. Due to the consistency rules, each variant sign has to be explained by a predecessor (Figure 8). that correspond to two different patient expression profiles, will predict an inhibition of JUN/FOS[n] with the sign consistency approach.

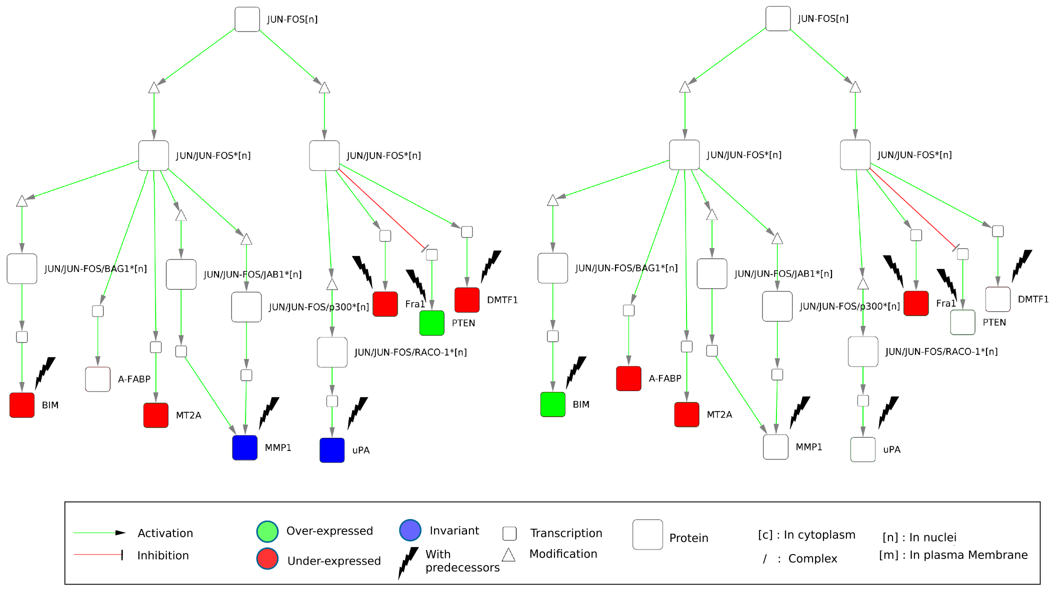

Figure 8: Subgraph from JUN/FOS[n] to variant genes with two examples of data coloring obtained from specific MM gene expression profiles. The graph is the same for each patient while the coloration is specific for each.

FOXM1 expression and survival

In order to analyze the correlation between *FOXM1* expression and the overall survival (OS), we selected the patients in the last quartile of *FOXM1* expression (Q4), with the highest level of expression and compared this group to the other quartiles (Figure 9). A log-rank test between these groups gave us a p-value <0.0001 allowing us to conclude to conclude to a correlation between better survival and low *FOXM1* expression.

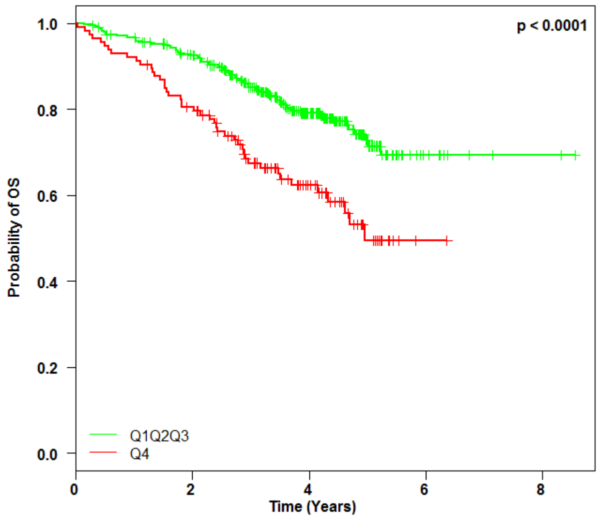

Figure 9: Overall survival (OS) in patients depending of *FOXM1* expression

Selected prognostic factors

In figure 10, we present the survival curves based on the couples identified as informative pronostic factors.

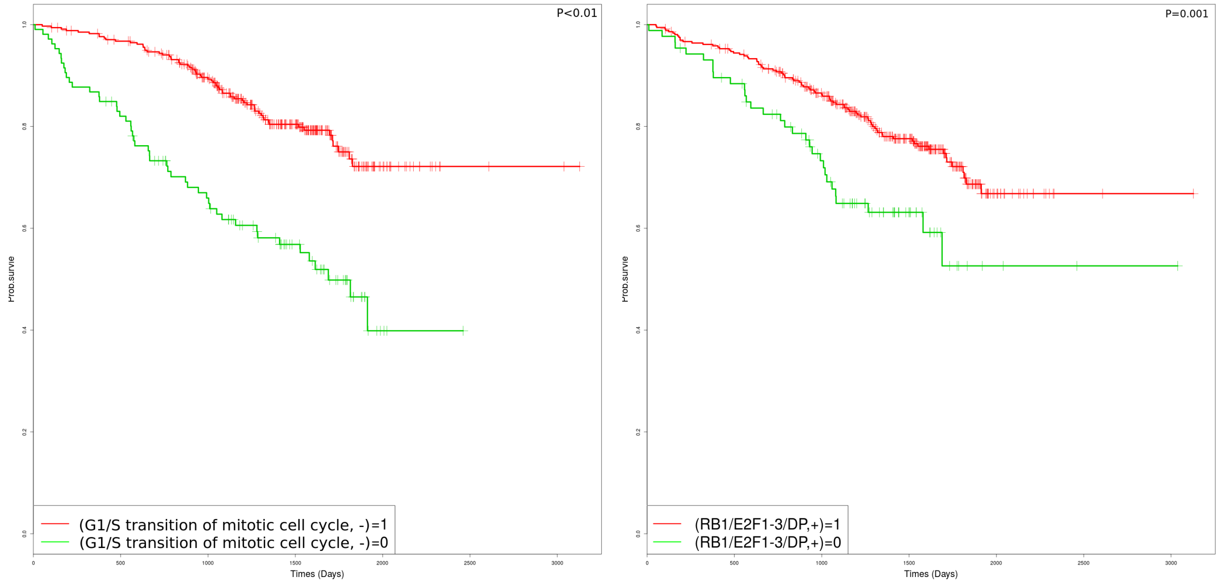

Figure 10: Overall survival (OS) in patients depending of inhibition of “G1/S transition of mitotic cell cycle” node (left) and inhibition of RB1/E2F1-3/DP[n] (right)

FOXM1C*[c] predictions and perturbation

In table 3, for FOXM1C*[c] and for 10 MC patients we indicate the prediction of the inhibition of this node (column Prediction(FOXM1C*[c],-), 1 if predicted, 0 if not) and the impact of the activation of FOXM1C*[c] (column Perturbation(FOXM1C*[c],+), 1 if the perturbation is in the 10% best perturbation for the dataset, 0 if not). The last column shows patients with both conditions : inhibition prediction of FOXM1C*[c] and activation of FOXM1C*[c] with a top-ranked SCENFIT scores. The first lines (sum and frequency) indicate respectively the sum and the frequency for each column for all MC patients.

Table 3: Comparison between predictions and perturbation for FOXM1 for an example of 10 MC patients

Patient	Prediction(FOXM1C*[c],-)	Perturbation(FOXM1C*[c],+)	Prediction(FOXM1C*[c],-) $\wedge$ Perturbation(FOXM1C*[c],+)
**Sum**	**466**	**219**	**212**
**Frequency**	**0.774**	**0.364**	**0.352**
MC1	1	1	1
MC2	0	0	0
MC3	0	0	0
MC4	1	1	1
MC5	1	0	0
MC6	1	1	1
MC7	1	0	0
MC8	1	1	1
MC9	0	0	0
MC10	1	0	0
